# Characterization of Mitochondrial Prohibitin in *Opsariichthys bidens* and Its Potential Functions in Spermatogenesis

**DOI:** 10.3390/ijms23137295

**Published:** 2022-06-30

**Authors:** Li Wang, Jingqian Wang, Xinming Gao, Chen Du, Congcong Hou, Chundan Zhang, Junquan Zhu, Daojun Tang

**Affiliations:** Key Laboratory of Applied Marine Biotechnology of Ministry of Education, Ningbo University, Ningbo 315832, China; w2424768530@163.com (L.W.); wangjingqian0527@163.com (J.W.); nbugxm4851@163.com (X.G.); 8788182@163.com (C.D.); houcongcong@nbu.edu.cn (C.H.); zhangchundan@nbu.edu.cn (C.Z.)

**Keywords:** prohibitin, mitochondria, spermatogenesis, *Opsariichthys bidens*

## Abstract

Spermatogenesis is the intricate and coordinated process by which spermatogonia develop into haploid differentiated spermatozoa. Mitochondria are essential for spermatogenesis, and prohibitin (PHB) is closely associated with mitochondrial structure and function during spermatogenesis. Although PHB has been implicated in spermatogenesis in some taxa, its roles in *Opsariichthys bidens* have not been determined. In this study, the expression patterns and potential functions of PHB in spermatogenesis in *O. bidens* were characterized using histological microscopic observations, PCR cloning, real-time quantitative PCR (qPCR), Western blotting (WB) and immunofluorescence (IF). The full-length cDNA of *Ob*-*phb* was 1500 bp encoding 271 amino acids. A sequence alignment demonstrated that the PHB protein is conserved among different animals. qPCR revealed that *phb* mRNA is widely distributed in *O. bidens* and highly expressed in the testes at stages IV and V. WB revealed that *Ob*-PHB is located in the mitochondria of testes. IF revealed the colocalization of PHB signals and mitochondria. Signals were detected around nuclei in spermatogonia and spermatocytes, gradually moving to the tail region during spermiogenesis, and finally aggregating in the midpiece. These results indicate that *Ob*-PHB was expressed in the mitochondria during spermatogenesis. In addition, this study proposed *Ob*-PHB may participate in the degradation of mitochondria and cell differentiation during spermatogenesis.

## 1. Introduction

Spermatogenesis in teleosts is a complex and dynamic developmental process involving a series of physiological and morphological changes in germ cells [1]. This process mainly includes three parts: mitotic divisions of spermatogonia, meiotic divisions of spermatocytes, and the transformation from spermatids to mature sperm [1]. Spermatogenesis is an energy-consuming process, in which the energy supply in the process of germ cell differentiation and sperm capacitation is directly related to ATP produced by mitochondria via oxidative phosphorylation [2]. Studies have shown that mitochondrial defects caused by environmental factors can disrupt spermatogenesis [3,4]. The mitochondria also participate in other processes that affect spermatogenesis. It can produce reactive oxygen species (ROS), which are beneficial for sperm capacitation and maturation but can cause oxidative stress and further damage sperm quality [5,6]. Mitochondrial DNA (mtDNA) mutations can cause meiosis arrest in spermatocytes and abnormal sperm morphologies [7,8]. In addition, mitochondrial proteins are closely related to sperm maturation [9,10]. For example, the silencing of glycerol-3-phosphate acyltransferase 2 (GPAT2) leads to sperm abnormalities [11]; this protein is important for de novo glycerolipid synthesis and normal spermatogenesis. Other studies of mammals have shown that the volume and number of mitochondria are positively associated with sperm morphology, fertilization ability, and sperm motility [12,13]. In most teleosts, mitochondria are essential for the maintenance of sperm motility [13].

Prohibitins (PHBs) are highly conserved proteins and widely distributed in bacteria [14], yeast [15], plants [16], and other species. The prohibitins gene family mainly comprises PHB and PHB2, both of which belong to the SPFH family, and they share a transmembrane, SPFH, and coiled-coil domains [17]. In eukaryotes, PHB1 and its homologous subunit PHB2 form heterodimers and are further assembled into an ordered PHB complex towards the mitochondrial intermembrane space [18,19]. The integrity of the PHB complex is the basis of its function; a lack of PHB not only damages mitochondria but also affects organism survival [20]. For example, in the yeast *Saccharomyces cerevisiae*, PHB mutations alter the morphology of mitochondria but do not lead to cell death [21,22], whereas the deletion of PHB homologues is fatal to the fruit fly *Drosophila* at the larval stage [23]. It has been proposed that PHB acts as an holdase/unfoldase chaperone to stabilize newly synthesized mitochondrial proteins [19], maintain the stability of mtDNA via mitochondrial transcription factor A (TFAM) [24,25], regulate the degradation of mitochondrial proteins by combining with the m-AAA proteasome [26] and promote mitochondrial fusion by interacting with optic atrophy 1 (OPA1) [27].

PHB is involved in spermatogenesis and has various biological functions. For instance, decreased PHB expression in *Bubalus bubalis* is correlated with low sperm motility [28]; a lack of PHB in *Caenorhabditis elegans* causes abnormal spermatogenesis [20]. In humans, PHB regulates sperm motility by affecting mitochondrial membrane potential (MMP) and ROS levels [29]. The role of PHB in the reproductive system has also been demonstrated in crustaceans [30,31,32]. In these species, PHB is highly expressed in spermatids, where it not only regulates mitochondrial degradation but also participates in nuclear shaping and acrosome formation [30,31,32]. In addition, in some teleosts such as the mudskipper *Boleophthalmus pectinirostris* and silver pomfret *Pampus argenteus*, PHB is expressed at all stages of spermatogenesis and maintains the normal morphology and function of mitochondria [33,34]. Therefore, these results promote an understanding of the relationship between PHB and spermatogenesis.

*Opsariichthys bidens* (Cypriniformes, Cyprinidae) is an important commercial fish in China and aquaculture production is increasing [35]. To develop artificial propagation technology for *O. bidens*, research on the mechanisms underlying reproduction in the species has attracted substantial attention. To date, cytological features of spermatogenesis in *O. bidens* have been studied by Tang et al. (2020) [35]. Spermatogenesis in *O. bidens* begins with the proliferation of spermatogonia, and after meiotic divisions of spermatocytes, haploid spermatids differentiate into flagellated sperm via morphological changes during spermiogenesis [35]. However, few studies have evaluated the molecular mechanisms of spermatogenesis in *O. bidens*. Based on the roles of PHB in reproduction and evolutionary conservation, we speculate that PHB plays an important role in *O. bidens* spermatogenesis.

In the present study, we observed the histological characteristics of testes and spermatogenic cells and explored the expression, subcellular localization and possible functions of PHB in *O. bidens*. PHB may play a role in maintaining the integrity and function of mitochondria and mediate mitochondrial degradation during fish spermatogenesis, laying the foundation for the elucidation of the mechanism of spermatogenesis in *O. bidens.*

## 2. Results

### 2.1. Histological Observations of Testis Development and Spermatogenesis in O. bidens

**Figure 1:**
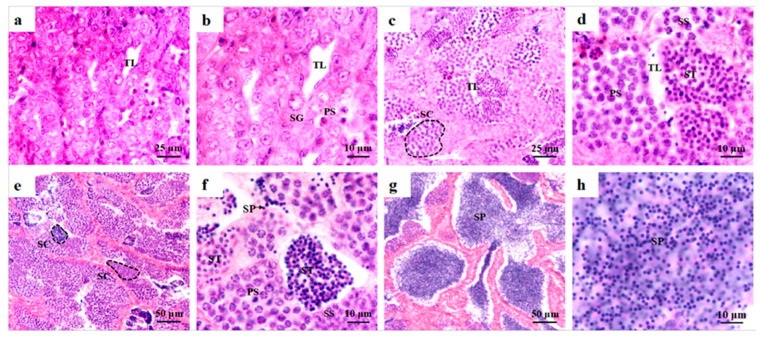
Histological observations of stage I–V testes in *O. bidens.* (**a**) Stage I testis. The seminiferous tubules have been formed with some small tubule lumens; (**b**) Enlarged image of (**a**). Spermatogonia and primary spermatocytes exist. (**c**) Stage III testis; (**d**) Enlarged image of (**c**). The testes are mainly composed of primary spermatocytes and include some of spermatogonia, secondary spermatocyte, spermatid. (**e**) Stage IV testis. Spermatids cysts increased, some sperm cysts burst, and spermatozoa flew into tubule lumen; (**f**) Enlarged image of (**e**). The testes are mainly composed of spermatocytes, spermatid and spermatozoa. (**g**) Stage V testis. Most sperm cysts opened, and spermatozoa were released into lumen of seminiferous tubule. (**h**) Enlarged image of (**g**). Mostly spermatozoa can be observed. SG: spermatogonia; PS: primary spermatocyte; SS: secondary spermatocyte; ST: spermatid; SP: sperm; SC: spermatogenic cyst; TL: tubule lumens.

**Figure 2:**
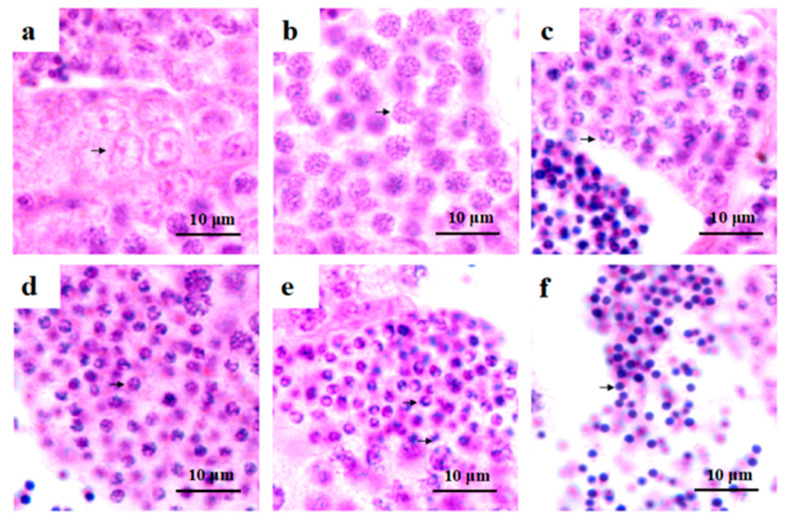
Histological observations of spermatogenesis in *O. bidens.* (**a**–**f**) Spermatogenic cells were processed by paraffinized sectioning. (**a**) Spermatogonia; (**b**) Primary spermatocyte; (**c**) Secondary spermatocyte; (**d**) Early spermatid; (**e**) Middle spermatid; (**f**) Sperm.

As shown in Figure 1, four stages of testis development were observed in *O. bidens* by histological analyzation. In stage II of testis development, testes were mainly composed of spermatogonia and included low amounts of primary spermatocytes. The seminiferous tubules were formed of some small tubule lumens. The spermatogonia were the main germ cells and were developed in the cysts and scattered in seminiferous tubules (Figure 1a,b). Spermatogonia were ovoid or spherical in shape with a large nucleus that contained prominent nucleoli (Figure 2a). In stage II of testis development, the testes were mainly composed of primary spermatocytes and included low amounts of spermatogonia, secondary spermatocytes, and spermatids, which developed in the cysts (Figure 1c,d). The primary spermatocytes (Figure 2b) developed from the spermatogonia and were relatively smaller in size with oval shapes. Following the first meiotic division, primary spermatocytes developed into secondary spermatocytes, which were smaller in size (Figure 2c). The nucleoli disappeared at the spermatocyte stage. In stage III of testis development, spermatid cysts increased, some sperm cysts burst, and spermatozoa entered into the tubule lumen (Figure 1e,f). The spermatids developed from secondary spermatocytes, in which the nuclear basophilia further increased and cells became smaller than secondary spermatocytes. The spermatids had different volumes and different degrees of chromatin condensation in various developmental stages. In the early development stage of spermatids (early spermatids), the cells were oval with round nuclei (Figure 2d). In the middle development stage of spermatids (middle spermatids), the cells were smaller, and the chromatin concentration was higher than that of early spermatids with increased basophilia (Figure 2e). In stage V of testis development, most of the sperm cysts opened and spermatozoa were released into the lumen of the seminiferous tubules (Figure 1g,h). The nuclei of spermatozoa were dark blue (Figure 2f).

### 2.2. Full-Length cDNA Cloning and Protein Structure Prediction

**Figure 3:**
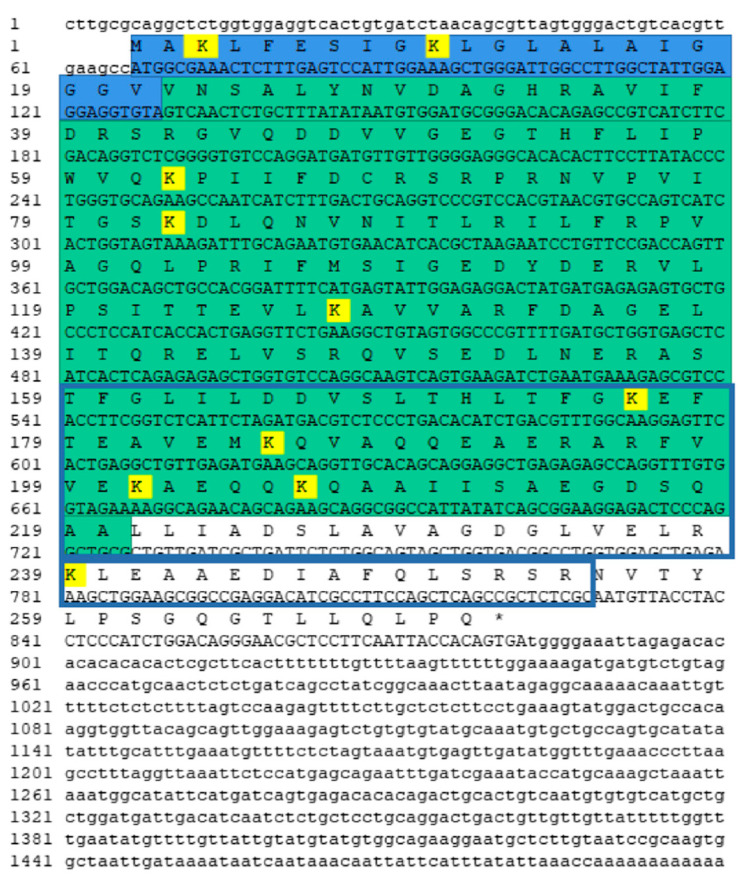
Full-length cDNA of *Ob-phb* and its amino acid sequence. The 5’ and 3’ UTR are marked with lowercase letters. Blue represents the transmembrane domain of the PHB protein, green represents the SPFH domain, and the blue rectangular area shows the predicted coiled-coil domain. Yellow indicates the conserved lysine sites in the PHB protein.

**Figure 4:**
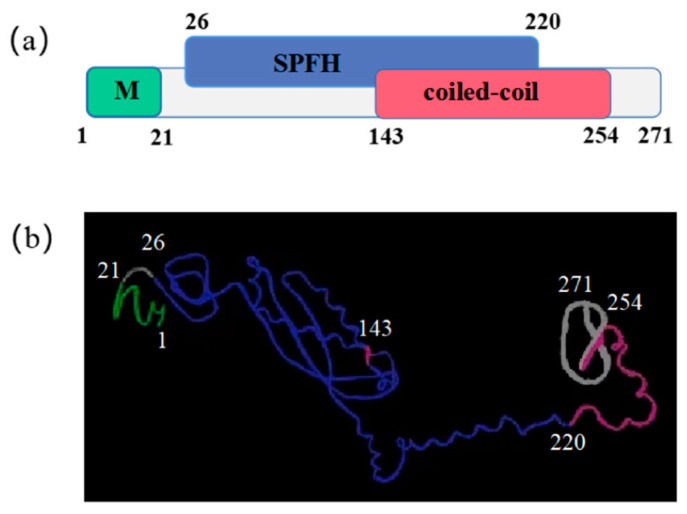
Putative domain structure and 3-D structure of *Ob*-PHB. (**a**) Green indicates the transmembrane domain (1–21 aa), blue indicates the putative PHB domain (26–220 aa), and red is the coiled-coil domain (143–254 aa). (**b**) Predicted 3-D structure of PHB.

The total length sequence of *Ob*-*phb* cDNA was 1500 bp, including 66 bp 5’ untranslated region (UTR), 816 bp open reading frame (ORF) and 618 bp 3’ UTR (Figure 3). The molecular weight of *Ob*-PHB was approximately 29.6 kDa and its isoelectric point was 5.16. The *Ob*-PHB protein contains 271 amino acids (aa) and its predicted secondary structure is shown in Figure 4a. It contained an N terminal hydrophobic stretch (from 1 aa to 21 aa), a central SPFH domain (from 26 aa to 220 aa), and a coiled-coil structure (from 143 aa to 254 aa). In addition, the tertiary structure of the *Ob*-PHB protein was predicted (Figure 4b).

### 2.3. Multiple Sequence Alignment and Phylogenetic Analysis

**Figure 5:**
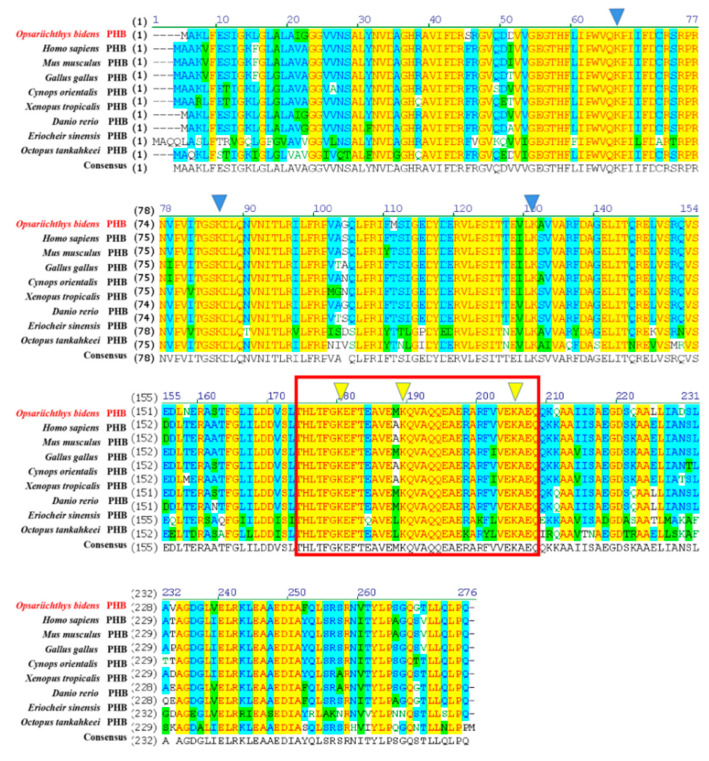
Sequence alignment of the PHB protein among *O. bidens* and other species. The red box shows highly conserved PHB fragments among species, and the yellow arrow marks the conserved tyrosine site. PHB protein sequence identities between *O. bidens* PHB and *H. sapiens*, *M. musculus*, *G. gallus*, *C. orientalis*, *X. tropicalis*, *D. rerio*, *E. sinensis*, and *O. tankahkeei* were 90.1%, 89.7%, 89.3%, 90.1%, 88.2%, 97.0%, 84.7% and 86.1%.

**Figure 6:**
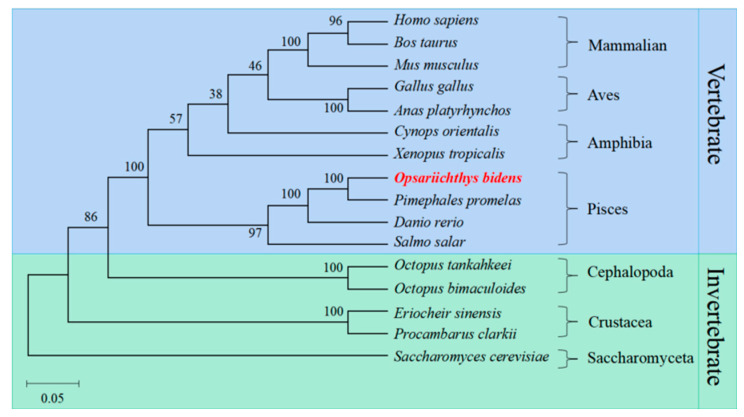
Phylogenetic tree based on PHB amino acid sequence. The putative *Ob*-PHB formed a sub-franch with fish PHB.

The *Ob*-PHB protein was compared with the homologous proteins of other species by a multiple sequence alignment and phylogenetic analysis (Figure 5). PHB protein sequence identities for *O. bidens* and *Homo sapiens*, *Mus musculus*, *Gallus gallus*, *Xenopus tropicalis*, *Cynops orientalis*, *Danio rerio*, *Eriocheir sinensis*, and *Octopus tankahkeei* were 90.1%, 89.7%, 89.3%, 88.2%, 90.1%, 97.0%, 84.7% and 86.1%, respectively. The phylogenetic tree included mammals, birds, amphibians, arthropods, fish, and other taxa, and *Ob*-PHB was closely related to homologues in *Pimephales promelas*, *Danio rerio*, and *Salmo salar* (Figure 6).

### 2.4. Expression Patterns of phb mRNA and PHB Protein

**Figure 7:**
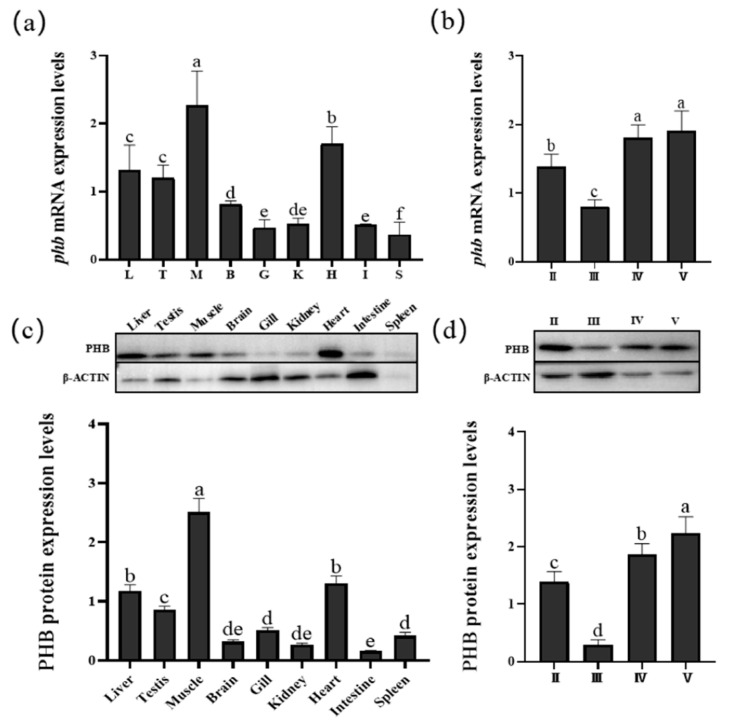
Expression pattern of *phb* mRNA and PHB protein. Expression pattern of *phb* mRNA and PHB protein in *O. bidens* in (**a**,**c**) various tissues and (**b**,**d**) in testes at stages II–V, as determined by WB and qPCR, with levels of β-actin as the positive control. I, III, IV, V indicate stages of testis development in *O. bidens*. L, T, M, B, G, K, H, I, and S indicate the liver, testis, muscle, brain, gill, kidney, heart, intestine, and spleen, respectively. The significance difference of data are represented by a–f. The same letter means that the difference is not significant (*p* > 0.05), and the different letter means that the difference is significant (*p* < 0.05).

We extracted RNA and protein from the liver, gonad, kidney, brain, gill, muscle, heart, intestine, and spleen and stages I–V testes of *O. bidens*, respectively. Subsequently, we performed qPCR and WB to analyze the expression levels of *phb* mRNA and PHB protein, respectively. As shown in Figure 7, *Ob-phb* mRNA and PHB protein were expressed in all of the tissues examined. Expression of the mRNA and protein was consistent in nine tissues and stage I–V testes, which were more expressed in the heart and muscle, moderately in the testes, and lowly in the kidney and intestine. In the testes, *Ob-phb* mRNA and PHB protein levels were highly expressed at stage IV and V.

### 2.5. Subcellular Localization and Distribution of the Ob-PHB Protein during Spermatogenesis

**Figure 8:**
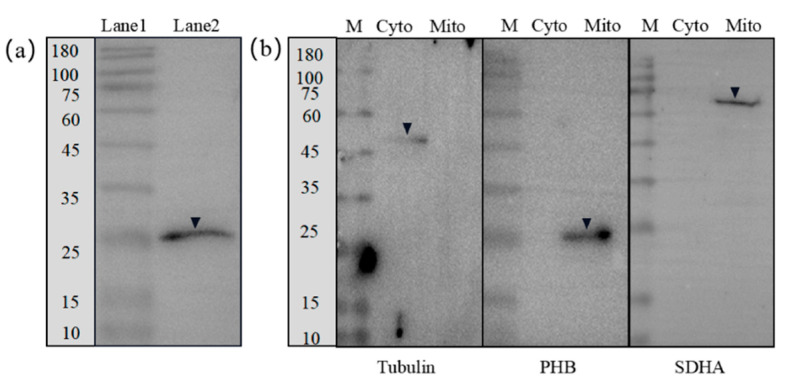
Western blotting images demonstrating *Ob*-PHB present in the mitochondria. (**a**) Western blotting images demonstrating the specificity of anti-PHB antibodies. Lane 1: protein marker; Lane 2: PHB protein. (**b**) Intracellular location of *Ob*-PHB in stage IV testes. SDHA was detected in Mito and β-tubulin was detected in Cyto. PHB was detected in Mito, confirming that it was located in the mitochondria. M: protein marker; Mito: mitochondrial protein fraction purified from the stage IV testis; Cyto: total protein extracted from the cytoplasmic fraction.

The specificity rabbit anti-PHB antibody (Beyotime, Shanghai, China) applied in WB was demonstrated (Figure 8a). WB results showed that *Ob*-PHB in spermatogenic cells was distributed in mitochondria. As shown in Figure 8b, SDHA was detected only in mitochondria. β-Tubulin was only detected in the cytoplasm. These results indicate that mitochondria were completely separated from the cytoplasm. Subsequently, we confirmed that PHB is only expressed in mitochondria.

**Figure 9:**
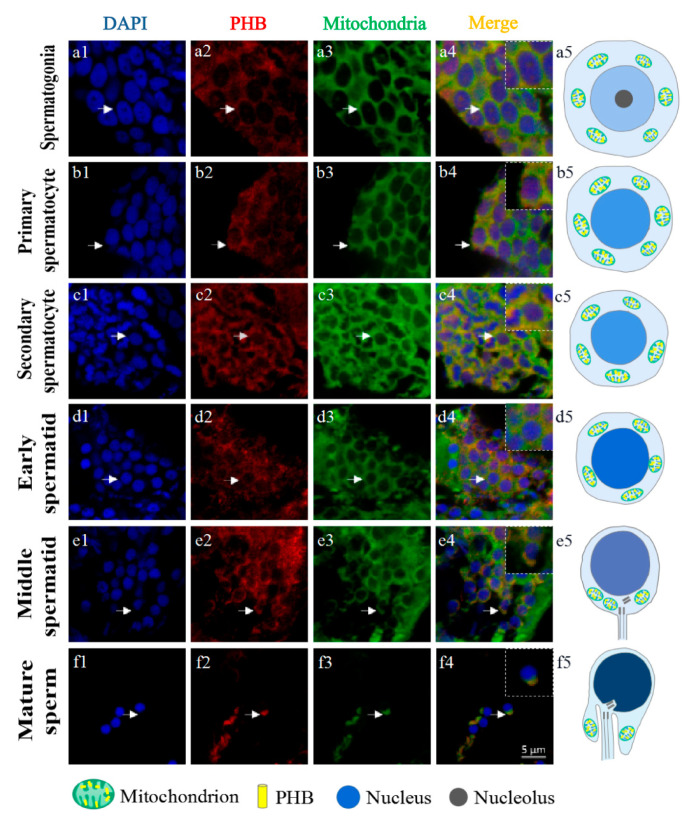
Immunofluorescence showed the distribution pattern of *Ob*-PHB and mitochondria during spermatogenesis. (**a5**,**b5**,**c5**,**d5**,**e5**) PHB and mitochondria distribution in different spermatogenic cells. In spermatogonia, spermatocytes and early spermatids (**a1**–**c5**), *Ob*-PHB and mitochondria signals were evenly distributed in the cytoplasm around the nuclei; in middle spermatids, signals shift to the tail region (**d1**–**d5**). In mature sperm, signals aggregated in the midpiece (**e1**–**e5**). White arrows refer to the colocalization signal of *Ob*-PHB and mitochondria. At the mature sperm stage, *Ob*-PHB and mitochondrial signals were only detected in the midpiece (**f1**–**f5**).

The WB experiment demonstrated that the rabbit anti-PHB antibody (Beyotime, Shanghai, China) can be used for IF (Figure 8a). In IF, the rabbit anti-PHB antibody can specifically bind to *Ob*-PHB protein acting as an antigen. IF was used to evaluate the expression and distribution of *Ob*-PHB during spermatogenesis. *Ob*-PHB signals were consistently co-localized with mitochondrial signals. In the spermatogonia and spermatocytes, *Ob*-PHB and mitochondrial signals were distributed around the nuclei (Figure 9(a1–c5)). In early spermatids, the distribution of *Ob*-PHB and mitochondrial signals was similar to that of spermatocytes (Figure 9(d1–d5)). In the middle spermatid, *Ob*-PHB and mitochondrial signals began to shift to the tail region (Figure 9(e1–e5)). At the mature sperm stage, *Ob*-PHB and mitochondrial signals were only detected in the midpiece (Figure 9(f1–f5)).

## 3. Discussion

### 3.1. Testis Development and Spermatogenesis in O. bidens

Testis development of the zebrafish *Danio* *rerio* were roughly classified into eight phases according to time period (i.e., each phase corresponds to one week of the reproductive cycle) [36]. According to morphological changes and characteristics of the spermatogenic cells in testes, the reproductive cycle in other fish of Cypriniformes, such as the folifer *Tor brevifilis* [37] and the grouper *Acrossocheilus fasciatus* [38] were divided into six stages: stages I–VI, also named as the primordial germ cell period, proliferating period, early growth, late growth, maturation and regressed, respectively [37]. Similarly, the reproductive cycle of *O. Bidens*, a species of Cypriniformes, is divided into four successive stages: stages I–V, stage I, and stage VI are not observed in *O. bidens*. In stage I, spermatogonia constantly proliferate and this can be referred to as the proliferating period. In stage III, the testis is dominated by spermatocytes in meiosis, which can also be r meferred to as the meiosis period, as described by Huszno et al. (2012) [36]. In stage IV–V, spermatids gradually differentiate into spermatozoa, also known as the spermiogenesis period [36]. Above all, the staging of *O. bidens* testis development provides evidence for the identification of the reproductive cycle of *O. bidens*.

With testis development, spermatogenesis was spontaneously activated. Spermatogenesis in teleosts are classified into two types: cystic and semi-cystic [39]. In the cystic type, when spermatogenic cells develop into spermatozoa, the spermatogenic cysts open and then spermatozoa are released into the tubule lumen, which has been demonstrated in the zebrafish *D. rerio* [40]. In the semi-cystic type, which occurs in few teleosts, the cysts open before spermatogenesis finished. Then, spermatogenic cells at different stages flow into tubule lumen and develop into mature sperm non-synchronously [39,41]. For example, in *Ophidion* SP. and *Bryconops affinis*, spermatogenic cysts burst when spermatids occurs [39,41]. Based on our results, the spermatogenesis in *O. bidens* belong to the cystic type, which is consistent with the report by Tang et al. (2020) [35].

Mitochondria are important for spermatogenesis; its localization in spermatogenic cells is associated with spermatogenic function [2,42]. In the early stage of spermatogenesis in some taxa, mitochondria are distributed around the nuclei [33,34]. In this stage, mitochondria may regulate metabolism levels by promoting cell proliferation and differentiation [42,43]. In addition, mitochondrial fusion is necessary for meiosis of spermatocytes [44]. During spermiogenesis, in some teleosts such as the silver pomfret *P. argenteus* and the mudskipper *B. pectinirostris*, mitochondria gradually shift to the area where flagellum formation occurs [33,34]. Mitochondria may provide energy for sperm motility and fertilization in this stage [33,34,43]. In this study, by IF, we detected mitochondrial signals throughout spermatogenesis in *O. bidens* (as shown in Figure 9). In *O. bidens*, mitochondria were distributed in cytoplasm in spermatogonia and spermatocytes (Figure 9(a1–c5)), while gradually shifted to the tail region and aggregated in the midpiece of the flagellum during spermiogenesis (Figure (d1–f5)), which is in agreement with reports by Gao et al. (2020). and Wang et al. (2017) [33,34]. Therefore, we deduce that mitochondria may promote cell proliferation and midpiece formation during spermatogenesis in *O. Bidens*.

### 3.2. High Conservation of PHB Protein

PHB is an important mitochondrial protein that was involved in animal spermatogenesis. In the present study, we retrieved the *Ob*-*phb* full-length cDNA and predicted its protein structure. An amino acid sequence alignment showed that PHB is highly homologous in many species, such as human, mice and zebrafish, indicating PHB may have conserved functions in animals (Figure 5). PHB is an important member of the SPFH family and typically contains the transmembrane PHB, a coiled-coil domain, as observed in PHB of the red crayfish *Procambarus clarkii* [29], the Chinese fire-bellied newt *C*. *orientalis* [32], the silver pomfret *P. argenteus* [33], and the mudskipper *B. pectinirostris* [34]. The *Ob*-PHB proteins share the same domain structure with other species (Figure 4). The N-terminal transmembrane domain contains a hydrophobic region consisting of about 20 amino acids (Figure 4), which may help anchor the PHB complex to the inner membrane of mitochondria [18], helping it function a membrane-bound chaperone to help newly synthesized peptide chains form the correct spatial configuration [19,24]. The C-terminal domain is very important for the formation of the prohibitin complex and the stability of the mitochondrial polypeptide chain [19,24,45]. Lysine in the coiled-coil domain makes PHB and PHB2 form a cross-linked peptide, which may further promote the formation of complete prohibitin complex [45]. Lysine acts as the interaction site as well as an important site of ubiquitination [45]. A sequence alignment revealed the coiled-coil domain of PHB has three conserved lysines. Thus, we can deduce that lysine in *Ob*-PHB promotes PHB complex formation. Because the primary and tertiary structures of PHB are evolutionarily conserved, we speculated that the function of *Ob*-PHB is also conserved.

### 3.3. PHB Is Widely Expressed in Organisms and Participates in Spermatogenesis

PHB is widely distributed in the Chinese fire-bellied newt *C. orientalis* [32], the silver pomfret *P. argenteus* [33] and many other species and has diverse functions, including functions in the regulation of cell proliferation and apoptosis [27]. Our qPCR and WB results demonstrated that PHB is expressed in all tissues of *O. bidens* at the mRNA and protein levels. Studies have shown that prohibitins are highly expressed in tissues that heavily rely on mitochondrial function [43]. Compared with levels in other tissues, *Ob*-PHB is highly expressed in the heart muscle, containing abundant mitochondria, consistent with results for the Chinese mitten crab *E. sinensis* [46], the Asian paddle crab *C. japonica* [31] and *O. tankahkeei* [47]. In addition, the expression levels of *Ob*-PHB in the testis was higher than in the brain, intestine, and spleen (Figure 7a,c). A previous study has showed that PHB in the Chinese fire-bellied newt *C. orientalis* is highly expressed in the testes, which constantly undergo mitosis and meiosis; it is possible that testes need abundant PHB, which exert certain influence in the reproductive cycle [32]. Another study has shown that the PHB expression levels in the testes of the mudskipper *B. pectinirostris* is the highest among various organs, suggesting that PHB is involved in spermatogenesis in the species [34]. Therefore, we propose that PHB may be involved in spermatogenesis in *O. bidens*. Further research revealed that *Ob*-PHB is expressed during different stages of testis development, with high expression levels in stages IV and V (Figure 7b,d). Mature spermatozoa are predominant in stage V testes, in which *Ob*-PHB may indirectly provide energy for sperm motility through mitochondria.

### 3.4. PHB May Influence Mitochondrial Structure and Function and Mediate Mitochondrial Degradation during Spermatogenesis in O. bidens

Extensive research has focused on the distribution of PHB. A previous study confirmed that PHB is a mitochondrial membrane protein, rather than a mitochondrial matrix protein in mammalian cells [48]. In the yeast *S. cerevisiae*, PHB and the m-AAA proteasome form a supercomplex that is localized on the mitochondrial inner membrane [26]. To explore the relationship between PHB and *O. bidens* spermatogenesis, we studied its location in *O. bidens* testes. WB results showed that PHB is located within mitochondria, rather than the cytoplasm extracted from Stage IV testis. Further IF experiments showed that *Ob*-PHB was always detected in mitochondria in spermatogenic cells of different stages, proving that PHB is located in the mitochondria and may exert a scaffolding function [24].

IF also showed that PHB is co-located with mitochondria and exists around the nucleus in spermatogonia and spermatocytes (Figure 9(a1–c5)). Some studies have shown that PHB are important for mitochondrial activity. The deletion of PHB caused abnormal mitochondria in *C. elegans* muscle cells and Hela cells [20,49]. Mitochondria are not only the main source of ROS but are also highly vulnerable to ROS [50]. Wang found that decreased PHB expression increases ROS levels, which may destroy the mitochondrial structure [51]. In addition, in human sperm, PHB can stabilize mitochondrial complex I, which is essential for the assembly of the oxidative phosphorylation (OXPHOS) system [52]. In the silver pomfret *P. argenteus* and the mudskipper *B. pectinirostris*, preliminarily analyses have shown that PHB is associated with mitochondrial activity during spermatogenesis [33,34]. Therefore, we deduced that *Ob*-PHB is involved in the maintenance of the integrity and function of mitochondria in spermatogonia and spermatocytes.

PHB is also very important for cell differentiation [53]. In human adipose-derived stem cells, a decrease in PHB expression inhibits adipocyte differentiation and restoring PHB contributes to adipocyte differentiation [54]. In rats, PHB is mainly expressed in non-proliferating thymocytes and may promote differentiation [55]. The processes of spermatogonia transformation into spermatocytes and spermatocyte transformation into spermatids all involve cell differentiation. Given the role of PHB in mitochondrial activity and cell differentiation, a deduction is that *Ob*-PHB may participate in spermatogenic cell differentiation by maintaining mitochondrial structure and function.

During spermiogenesis, PHB signals gradually move to the tail region (Figure 9(d1–d5)), and in mature sperms, PHB signals are located in the midpiece (Figure 9(e1–e5)). Jin et al. found that PHB in the Chinese fire-bellied newt *C. orientalis* can be ubiquitinated and may mediate mitochondrial degradation before and after fertilization to ensure maternal inheritance [32]. Similar conclusions were also reported in the red crayfish *P. clarkii* and the Chinese mitten crab *E. sinensis* [29,46]. Therefore, we hypothesize that *Ob*-PHB is involved in mitochondrial degradation during spermiogenesis.

## 4. Materials and Methods

### 4.1. Animal Preparation

From September 2020 to June 2021, *O. bidens* were sampled from the Huanya Aquaculture Company in Ningbo City, Zhejiang province. Approximately 10 experimental subjects were dissected per month to obtain tissues. The gonads were divided into two parts: one part was fixed in Bonn’s solution and paraffin sectioning was performed, and one part was fixed in 4% PFA-PBS and frozen sectioning was performed for subsequent Immunofluorescence (IF) experiments. In addition, nine tissues, including the liver, testes, kidney, brain, gill, muscle, heart, intestine, and spleen were obtained, frozen in liquid nitrogen and then stored at −80 °C until subsequent RNA and protein extraction.

### 4.2. Histological Analysis

Paraffin sectioning and hematoxylin-eosin staining was performed to observe the stages of testis development and the cytological characteristics of spermatogenesis. In brief, the gonad fixed with Bouin’s solution dehydrated by an ethanol concentration gradient, permeabilized with xylene, and embedded in paraffin. Then, samples were cut into 7 μm sections and stained with hematoxylin-eosin. Following paraffin sectioning completed, a Nikon NI-U light microscope (Nikon, Tokyo, Japan) was used to select the male *O. bidens* and to observe the stages of testis development and the cytological characteristics of spermatogenesis and, finally, images were obtained.

### 4.3. Full-Length cDNA Cloning of Ob-phb 

RNA was extracted from testes in *O. Bidens*. RNA extraction was performed as previously described [56]. First, tissue samples were mixed with TRIzol Reagent (Invitrogen, Carlsbad, CA, USA) and homogenized. Then, chloroform, isopropanol (to precipitate RNA), and 75% ethanol were added to isolate RNA. RNA was stored at −80 °C until use for reverse transcription. Then, RNA was reversed transcribed by using a PrimeScript^®^ RT Reagent Kit (Takara, Beijing, China). Correspondingly, the 5’ and 3’ RACE cDNAs were synthesized by reverse transcription using the Smart RACE cDNA Amplification Kit (ClonTech, Mountain View, CA, USA) with RNA extracted before as the template.

Based on the *Ob*-*phb* transcript obtained from the National Center for Biotechnology Information (https://www.ncbi.nlm.nih.gov/, accessed on 1 November 2021, NCBI), we designed primers using Primer Premier version 5.0 (Premier Biosoft In- 140 ternational, Palo Alto, CA, USA) (as shown in Supplementary file). Touch-down PCR was conducted to achieve the intermediate fragment of *Ob*-*phb* under the following program: 94 °C for 5 min, 8 cycles of 94 °C for 30 s, 56 °C for 30 s, and 72 °C for 50 s (decreased by 0.5 °C per cycle); 27 cycles of 94 °C for 30 s, 52 °C for 30 s, and 72 °C for 50 s; and 72 °C for 10 min for a final extension. Then, purpose straps were separated by performing gel electrophoresis and recycled using an Agarose gel Recovery Kit II (Bioteke, Beijing, China). Then, the purified cDNA product was inserted into the pMD19-T vector (Takara, Kusatsu, Japan) to form recombinant plasmids, which were transferred into competent DH5α cells (AngYuBio, Shanghai, China) and sent to GENEWIZ company for sequencing.

After the intermediate *Ob*-*phb* cDNA fragment was obtained, specific primers for 5’ and 3’ RACE were designed (Table 1). The nested PCR (two rounds) procedure for 5’ RACE was conducted as follows: 94 °C for 5 min, 8 cycles of 94 °C for 30 s, 69.5/64 °C for 30 s, and 72 °C for 60 s (decreased by 0.5 °C per cycle); 27 cycles of 94 °C for 30 s, 65.5/60 °C for 30 s, and 72 °C for 60 s; 72 °C for 10 min for a final extension. Subsequent steps were identical to those used for intermediate fragment cloning. After assembling intermediate fragment and 5′/3′ cDNA sequences, we obtained the full-length cDNA of *Ob*-PHB.

### 4.4. Analysis and Prediction of PHB Protein

Vector NTI 11.5 (Invitrogen, CA, USA) was used to determine the similarity between the PHB amino acid sequences of *O. bidens* and other species, and a multiple sequence alignment was constructed. A phylogenetic analysis was conducted by using MEGA 5.1 (Mega Limited, Auckland, New Zealand) with the neighbor-joining method. PHB from the following species (and accession numbers) included in the analysis were as follows: *H. sapiens* (AAB21614.1), *B. taurus* (NP_001029744.1), *M. musculus* (NP_032857.1), *G. gallus* (NP_001171206.1), *A. platyrhynchos* (XP_027301386.1), *C. orientalis* (AJF36071.1), *X. tropicalis* (NP_989038.1), *P. Promelas* (XP_039543669.1), *D. rerio* (NP_958454.1), *S. salar* (XP_014059527.1), *O. tankahkeei* (AEI91930.1), *O. bimaculoides* (XP_014779632.1), *E. sinensis* (ADM64319.1), *P. clarkii* (AGU02225.1) and *S. cerevisiae* (AAA53144.1). The isoelectric point and the molecular weight were predicted using ExPASy ProtParam (https://www.expasy.org/, accessed on 25 December 2021). COILS (http://coiledcoils.chm.bris.ac.uk/LOGICOIL/, accessed on 25 December 2021) was used to predict the coiled-coil domain in PHB, and TMpred (https://embnet.vital-it.ch/software/TMPRED_form.html, accessed on 25 December 2021) was used to predict the transmembrane domain. CD-search (https://blast.ncbi.nlm.nih.gov/Blast.cgi, accessed on 25 December 2021) was used to search conserved domains (SPFH domain) in PHB. In addition, the tertiary structure of the PHB protein was predicted by the online tool I-Tasser (https://zhanglab.ccmb.med.umich.edu/I-TASSER/, accessed on 25 December 2021).

### 4.5. Real-Time Quantitative PCR

We extracted mRNA from nine tissues of three male O. bidens (testes at stage IV) and testes of twelve male O. bidens (three individuals in each stage). Then, cDNA was synthetized using a PrimeScript® RT Reagent Kit (Takara, Beijing, China) for qPCR. We performed qPCR to evaluate phb mRNA expression levels in all of the tissues examined and stage II–V testes of O. bidens as described by Lin et al. [57]. According to the cloned full-length Ob-phb cDNA, specific primers and primers for β-actin (internal reference) were designed with the conditions of 80–150 bp between primers, 17–25 bp primer lengths, and 45–55% GC (Supplementary file). Primers are shown in Supplementary file, and cDNA was used as a template for PCR using the Roche LightCycler 480 Real-time Fluorescence Quantitative PCR instrument and SYBR green probe. The qPCR amplification was run as follows: 95 °C for 5 min, followed by 40 cycles (95 °C for 15 s, 60 °C for 15 s, 72 °C for 10 s). With the kidney as the comparator samples, the relative Ob-phb quantitative expression levels were analyzed by the 2^−ΔΔCT^ method and expressed as the mean ± SD (standard deviation; n = 3). The one-way analysis of variance (ANOVA) with SPSS v20.0 (IBM, Amonk, NY, USA) was used to determine significant differences. *p* < 0.05 was considered a statistically significant difference.

### 4.6. Western Blotting

The samples used for WB were the same as described in qPCR. Protein extraction assay was performed based on Zhang’s research [56]. First, RIPA (Solarbio, Shanghai, China) was used to extract the total protein from each tissue, with PMSF as a protease inhibitor. Second, the mixture was homogenized and centrifuged. Then, the supernatant was mixed with 5× SDS protein loading buffer at a ratio of 1:4, denatured in boiling water for 10 min, and stored at −20 °C.

WB assay was used to detect the expression pattern of Ob-PHB protein. WB experiments were performed according to Zhang’s research [56]. In the WB experiment, the denatured proteins were loaded in 10% gels and transferred to a PVDF membrane (Bio-Rad, Hercules, CA, USA). Then, the membrane was incubated with the primary antibody at 4 °C overnight and secondary antibody at 37 °C for 1 h in turn. The primary antibody was a rabbit anti-PHB antibody (Beyotime, Shanghai, China; 1:500 dilution), the secondary antibody was HRP-conjugated goat anti-rabbit IgG (Beyotime, Shanghai, China; 1:1500 dilution). Finally, the signals were detected using a chemiluminescence imager (Tanon 5200; Shanghai, China) and images were analyzed using image J (National Institutes of Health, Bethesda, MD, USA). The relative PHB protein levels were presented as mean ± SD and analyzed using one-way analysis of variance (ANOVA), and the statistically significant difference of expressions was defined as *p* < 0.05.

### 4.7. Extraction of Mitochondria and Detection of the PHB Protein

Mitochondria and cytoplasm were extracted from stage IV testes with the tissue Mitochondria Isolation Kit (Beyotime, Shanghai, China), according to the manufacturer’ instructions. In the WB experiment, the mitochondrial protein succinate dehydrogenase (SDHA) and cytoplasmic protein β-tubulin were used to confirm the separation between the mitochondria and cytoplasm. *Ob*-PHB protein was detected in the mitochondria and cytoplasm by WB. Rabbit anti-SDHA and rabbit anti-β-tubulin antibodies were purchased from Beyotime (Shanghai, China).

### 4.8. Immunofluorescence

Frozen sectioning was performed as follows: *O. bidens* testes were fixed in 4% PFA-PBS overnight. Following washing in PBS and after being dehydrated in 0.5 M sucrose-PBS at 4 °C, the testis was embedded in an optimum cutting temperature (O.C.T.) compound and stored at −80 °C. Then, samples were sliced into 5 μm frozen sections.

IF assay was conducted according to Zhang’s research [56]. For IF, frozen testis sections were supplemented with 0.3% Triton-X-100-PBS and blocked with 5% BSA, followed by incubation with the primary antibody rabbit anti-PHB antibody (1:80) at 4 °C overnight. Then, sections were incubated with the second antibody Alexa Fluor 555-labeled goat anti-rabbit IgG (h+l) (1:500), MitoTracker Deep Green FM (1:2000) at 37 °C for 1 h, respectively. Finally, the results were observed, and images were obtained using a laser confocal microscope (LSM880; Carl Zeiss, Oberkochen, Germany).

## 5. Conclusions

In this study, we analyzed the histological characteristics of testes and germ cells in *O. bidens* and explored the structure, distribution and functions of mitochondrial protein PHB during spermatogenesis. As determined by qPCR and WB, PHB was expressed in different organs and during different stages of testis development. IF showed that PHB and mitochondria are co-localized during spermatogenesis. These results indicated that PHB plays different roles in different stages of spermatogenesis. It may not only affect the structure, function, and degradation of mitochondria but also participate in the differentiation of spermatogenic cells. However, the exact mechanism by which PHB participates in spermatogenesis in *O. bidens* is still unknown and should be explored in further research.

## Figures and Tables

**Table 1:** The primer and probe used in this study for *phb* cDNA full-length cloning.

Primer/Probe	Sequence (5′-3′)	Purpose
PHBF1	TTTGTGGTAGAAAAGGCAG	PCR
PHBF2	AGTTTTCTTGCTCTCTTCCT	PCR
PHBR1	GTGGTAATTGAAGGAGCG	PCR
PHBR2	ATTAGCCACTTGCGGAT	PCR
3′PHBF1	CTCGCAATGTTACCTACCTCCC	3′ RACE
3′PHBF2	GGAAAAGATGATGTCTGTAGAACCC	3′ RACE
3′PHBF3	TCTTGCTCTCTTCCTGAAAGTATGG	3′ RACE
5′PHBR1	GGTAGGTAACATTGCGAGAGCGG	5′ RACE
5′PHBR2	CCTGCTTCTGCTGTTCTGCCTTTT	5′ RACE
5′PHBR3	AGATGGGAGGTAGGTAACATTGCGA	5′ RACE
qPCR-F	GAAGCCAATCATCTTTGACTGC	qPCR
qPCR-R	TCGGAACAGGATTCTTAGCGT	qPCR
β-actin F	TCCGTGACATCAAGGAGAAGC	qPCR
β-actin R	GGCAACGGAAACGCTCATT	qPCR

## Data Availability

The authors declare that all of the data supporting the findings of this study are available within the article.

## Supplementary Materials

The following supporting information can be downloaded at: https://www.mdpi.com/article/10.3390/ijms23137295/s1.
