# Supplementary material for: Characterization of Mitochondrial Prohibitin in Opsariichthys bidens and Its Potential Functions in Spermatogenesis
Source: Int J Mol Sci. 2022 Jun 30;23(13):7295. doi: 10.3390/ijms23137295 (PMC9266877; doi:10.3390/ijms23137295)
Supplement: Supplementary file 1 [file ijms-23-07295-s001.zip › ijms-1770906-supplementary.pdf]

1 cttgcgcaggctctggtggaggtcactgtgatctaacagcgttagtgggactgtcacgtt

1 M A K L F E S I G K L G L A L A I G

61 gaagccATGGCGAAACTCTTTGAGTCCATTGGAAAGCTGGGATTGGCCTTGGCTATTGGA

19 G G V V N S A L Y N V D A G H R A V I F

121 GGAGGTGTAGTCAACTCTGCTTTATATAATGTGGATGCGGGACACAGAGCCGTCATCTTC

39 D R S R G V Q D D V V G E G T H F L I P

181 GACAGGTCTCGGGGTGTCCAGGATGATGTTGTTGGGGAGGGCACACACTTCCTTATACCC

59 W V Q K P I I F D C R S R P R N V P V I

241 TGGGTGCAGAAGCCAATCATCTTTGACTGCAGGTCCCGTCCACGTAACGTGCCAGTCATC

79 T G S K D L Q N V N I T L R I L F R P V

301 ACTGGTAGTAAAGATTTGCAGAATGTGAACATCACGCTAAGAATCCTGTTCCGACCAGTT

99 A G Q L P R I F M S I G E D Y D E R V L

361 GCTGGACAGCTGCCACGGATTTTCATGAGTATTGGAGAGGACTATGATGAGAGAGTGCTG

119 P S I T T E V L K A V V A R F D A G E L

421 CCCTCCATCACCACTGAGGTTCTGAAGGCTGTAGTGGCCCGTTTTGATGCTGGTGAGCTC

139 I T Q R E L V S R Q V S E D L N E R A S

481 ATCACTCAGAGAGAGCTGGTGTCCAGGCAAGTCAGTGAAGATCTGAATGAAAGAGCGTCC

159 T F G L I L D D V S L T H L T F G K E F

541 ACCTTCGGTCTCATTCTAGATGACGTCTCCCTGACACATCTGACGTTTGGCAAGGAGTTC

179 T E A V E M K Q V A Q Q E A E R A R F V

601 ACTGAGGCTGTTGAGATGAAGCAGGTTGCACAGCAGGAGGCTGAGAGAGCCAGGTTTGTG

199 V E K A E Q Q K Q A A I I S A E G D S Q

661 GTAGAAAAGGCAGAACAGCAGAAGCAGGCGGCCATTATATCAGCGGAAGGAGACTCCCAG

219 A A L L I A D S L A V A G D G L V E L R

721 GCTGCGCTGTTGATCGCTGATTCTCTGGCAGTAGCTGGTGACGGCCTGGTGGAGCTGAGA

239 K L E A A E D I A F Q L S R S R N V T Y

781 AAGCTGGAAGCGGCCGAGGACATCGCCTTCCAGCTCAGCCGCTCTCGCAATGTTACCTAC

259 L P S G Q G T L L Q L P Q *

841 CTCCCATCTGGACAGGGAACGCTCCTTCAATTACCACAGTGAtggggaaattagagacac

901 acacacacactcgcttcactttttttgttttaagtttttttggaaaagatgatgtctgtag

961 aacccatgcaactctctgatcagcctatcggcaaacttaatagaggcaaaaacaaattgt

1021 ttttctctcttttagtccaagagttttcttgctctcttcctgaaagtatggactgccaca

1081 aggtggttacagcagttggaaagagtctgtgtgtatgcaaatgtgctgccagtgcatata

1141 tatttgcatttgaaatgttttctctagtaaatgtgagttgatatggtttgaaacccttaa

1201 gcctttaggttaaattctccatgagcagaatttgatcgaaataccatgcaaagctaaatt

1261 aaatggcatattcatgatcagtgagacacacagactgcactgtcaatgtgtgtcatgctg

1321 ctggatgattgacatcaatctctgctcctgcaggactgactgttgttgttatttttggtt

1381 tgaatatgttttgttattgtatgtatgtggcagaaggaatgctcttgtaatccgcaagtg

1441 gctaattgataaaataatcaataaacaattattcatttatattaaaccaaaaaaaaaaaa
